# Fabrication of Cellulose Nanofiber/AlOOH Aerogel for Flame Retardant and Thermal Insulation

**DOI:** 10.3390/ma10030311

**Published:** 2017-03-17

**Authors:** Bitao Fan, Shujun Chen, Qiufang Yao, Qingfeng Sun, Chunde Jin

**Affiliations:** 1 School of Engineering, Zhejiang A&F University, Hangzhou 311300, China; 18357178962@163.com (B.F.); 18868196155@163.com (S.C.); yaoqiufang105@163.com (Q.Y.); 2 Key Laboratory of Wood Science and Technology, Hangzhou 311300, China

**Keywords:** aluminum oxyhydroxide, cellulose nanofiber, flame retardant, thermal insulation

## Abstract

Cellulose nanofiber/AlOOH aerogel for flame retardant and thermal insulation was successfully prepared through a hydrothermal method. Their flame retardant and thermal insulation properties were investigated. The morphology image of the cellulose nanofiber/AlOOH exhibited spherical AlOOH with an average diameter of 0.5 μm that was wrapped by cellulose nanofiber or adhered to them. Cellulose nanofiber/AlOOH composite aerogels exhibited excellent flame retardant and thermal insulation properties through the flammability test, which indicated that the as-prepared composite aerogels would have a promising future in the application of some important areas such as protection of lightweight construction materials.

## 1. Introduction

Flame retardant materials are used increasingly and extensively in industry, agriculture and daily life owing to their good mechanical properties [1], fire resistance [2], and chemical stability. However, their applications are limited because many of them are non-degradable materials. The inorganic AlOOH particle is one of the most widely used inorganic flame retardant additives due to its demonstrated properties such as being tasteless, non-toxic, heat resistance, and non-volatile. It decomposes at 400 °C [3] according to the following reaction [4]:

2AlOOH (s) → Al_2_O_3_ (s) + H_2_O (g) (1)

Cellulose is considered as an almost inexhaustible source of raw material for the increasing demand for environmentally friendly [5] and biocompatible products [6,7]. Interest has recently arisen in the nanometer-size cellulose due to its unique characteristics such as the nanoscale dimensions [8], high surface area [9], unique morphology [10], low density [11], and high mechanical strength [12]. A great number of applications of cellulose nanofiber (CNF) are undertaken such as the biodegradable packaging materials [13], reinforcement components [14], and oxygen-barrier layers [15]. However, the intrinsic inflammability of cellulose makes it impossible to be used in many important areas such as lightweight construction. Therefore, the flame retardant modification of CNF aerogel is crucial. The main obstacle is how to realize fine dispersion of flame retardants in the CNF and avoid the collapse with the addition of abundant inorganic fillers.

Pristine cellulose nanofibers alone or in combination with other nanomaterials show very interesting physical and chemical properties that open up new potential application fields as functional materials. The research of inorganic nanoparticles/cellulose composite materials is currently a very exciting research area in fields such as sensor [16], electrical [17,18], magnetic [19], and optical properties [20,21]. In the present paper, cellulose nanofiber/AlOOH (CNFA) aerogel for flame retardant and thermal insulation was successfully fabricated through a facile hydrothermal method. Instead of the traditional mixing of flame retardants in a polymer matrix, in the system of CNFA, AlOOH was generated and wrapped in the CNF or adhered to the fibers. CNF acted as a scaffold in order to avoid the agglomeration of AlOOH. Combining cellulose and AlOOH could be considered as an effective and competitive route to obtain lightweight composites with outstanding flame retardant and thermal insulation performance.

## 2. Materials and Methods

### 2.1. Materials

The aluminum sulfate octadecahydrate (Al_2_(SO_4_)_3_·18H_2_O), urea (CO(NH_2_)_2_), and other chemicals were analytical grade and produced from Kermel Chemical Reagent Co., Ltd., Hangzhou, Zhejiang, China. The bamboo was obtained from the bamboo forest in Zhejiang, China. All the chemicals were used as received. Deionized water was used in all experiments.

### 2.2. Preparation of CNF Colloid Solution

Purified cellulose extracted from bamboo was referred to Xie et al. [22]. Typically, 2 g of purified cellulose was soaked in 1000 mL deionized water and split into thinner fibrils through ultrasonic treatment. Sonication was performed at 60 kHz with a 25-mm-diameter titanium horn under a 50% duty cycle (i.e., a repeating cycle of 0.5 s ultrasonic treatment and 0.5 s shutdown) in an ice bath. The sonication was conducted for 30 min with an output power was 300 W. CNF colloid solution with the weight concentration 0.2 wt %) was thus obtained.

### 2.3. Preparation of CNF and CNFA Aerogels

Then 1 mmol Al_2_(SO_4_)_3_ and 8 mmol CO(NH_2_)_2_ were added in 70 mL of the solution of CNF aqueous solution under magnetic stirring for 15 min at room temperature. Subsequently, it was transferred and sealed into a Teflon-lined stainless steel autoclave, heated at 100 °C for 8 h. The suspension was cooled down naturally and washed by a centrifuge treatment with 6000 rpm/s for 5 min. To obtain the homogeneous solution, the bulk after centrifuge treatment was put into 100 mL deionized water, and then underwent an ultrasonic treatment for 1 min with an output power of 50 W in an ice water bath to disperse the solution. The obtained suspension of CNFA and CNF solution obtained before were separated into moulds and placed in a refrigerator for about 12 h. The frozen samples were freeze-dried for 60 h using a Scientz-18N freeze-dryer to sublime the materials. The cold trap temperature was below −55 °C, and the vacuum pressure was below 10 μPa during the freeze-drying process. The lightweight, solid, and sponge-like CNF and CNFA aerogels were fabricated successfully and used for characterization.

### 2.4. Characterization

The morphological features of the CNF and CNFA were characterized by scanning electron microscope (SEM, FEI, Quanta 200, Hillsboro, OR, USA) and transmission electron microscope (TEM, FEI, Tecnai G20, Hillsboro, OR, USA). The SEM images were obtained operated with 12.5 kV acceleration voltages, 5 mm working distance, and using an auto fine coater to coat the samples with gold to improve the conductivity. The sample used for TEM was prepared as follows: drops of dilute cellulosic network suspensions were deposited into glow-discharged carbon-coated TEM grids. The excess liquid was absorbed by a piece of filter paper. After the specimen has been completely dried, it was observed operated at 80 kV. The size distribution was analyzed by the software of Nano Measurer 1.2.5 (Fudan University, Shangshai, China). Density of the aerogel was calculated by the eq. (m/ν), where m was the mass of the sample and ν was the volume. Porosity of the aerogel was calculated according to the eq (1 − ρ*/ρ) × 100%, where ρ* was the density of the aerogel and ρ was theoretical density of cellulose [23]. Crystalline structures were identified by X-ray diffraction technique (XRD, Rigaku, D/MAX 2200, Tokyo, Japan) operating with Cu Kα radiation (λ = 1.5418 Å) at a scan rate (2θ) of 4°·min^−1^ and the accelerating voltage of 40 kV and the applied current of 30 mA ranging from 5° to 80°. The X-ray photoelectron spectroscopy (XPS, ULVAC–PHI, Inc., Chigasaki, Japan) analysis of the specimen was carried out using a microfocused (100 mL, 25 W, 15 kV) monochromatic Al-K Kα source (hm = 1486.6 eV), a hemispherical analyzer, and a multichannel detector. FTIR spectra were recorded on a Fourier transform infrared (FTIR) instrument (Nicolet Magna 560, Thermo Electron Corp., Madison, WI, USA) in the range of 400–4000 cm^−1^ with a resolution of 4 cm^−1^. The samples were ground into powder by a fiber microtome and then blended with KBr before pressing the mixture into ultra-thin pellets. The thermal conductivity of aerogels was measured by the transient plane source method (Hot Disk 2500, Uppsala, Sweden).

## 3. Results and Discussion

**Figure 1:**
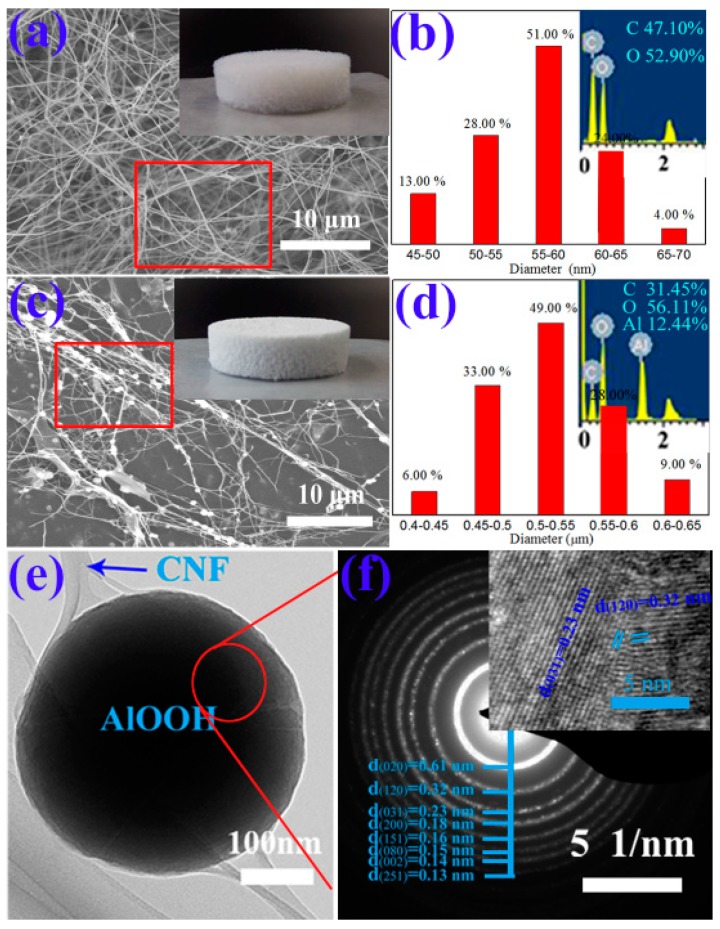
The morphological features of cellulose nanofiber (CNF) and cellulose nanofiber/AlOOH (CNFA). (**a**) Scanning electron microscope (SEM) micrographs of CNF and the macroscopic morphology of CNF aerogel in the insert; (**b**) The corresponding diameter distributions of the CNF and inset was corresponding the EDS spectra; (**c**) SEM image of CNFA and the macroscopic morphology of CNFA aerogel in the insert; (**d**) The corresponding diameter distributions of the CNFA and inset was corresponding the EDS spectra; (**e**) The enlarged transmission electron microscope (TEM) image of CNFA; (**f**) SAED of CNFA and HRTEM image in the insert.

The morphological features of CNF and CNFA are shown in Figure 1. As shown in Figure 1a, the microstructure of CNF was plentiful slender fibrils with widths of 45–70 nm (Figure 1b). It was clear that the average diameter of the fibers was about 56.3 nm. The macroscopic morphology of CNF aerogel was white with well-defined shape in the insert of Figure 1a. The density of the CNF aerogel was 2 mg·cm^−3^, and the porosity was 99.88%. In the insert of Figure 1b, the peaks of C and O were observed in the EDS spectra of the samples, indicating the constituent elements of CNF. Figure 1c showed the SEM image of CNFA, the shape was showed in the insert. The density and the porosity of the CNFA composite aerogel was 2.5 mg·cm^−3^ and 99.85%. After the hydrothermal reaction, spherical AlOOH was generated and wrapped in the CNF or adhered to the fibers. The particle size distribution was showed in Figure 1d. The AlOOH was spherical with the diameter of 0.4–0.65 μm. It was clear that the average diameter of the spherical AlOOH was 0.5 μm. The Al element peak was present in the EDS spectra of CNFA, which suggested that the Al element was sequestrated during the hydrothermal reaction. Figure 1e showed the enlarged TEM image of CNFA, it can be observed that a spherical AlOOH particle was wrapped by CNF. Figure 1f showed the SAED pattern of a selected area. Eight main diffraction rings correspond to the (020), (120), (031), (200), (151), (080), (002), and (251) planes of polycrystalline orthorhombic AlOOH. The distribution of orthorhombic AlOOH particles was displayed by the HRTEM image in the insert of Figure 1e. The results clearly reveal lattice fringe with a d-spacing of 0.32 nm and 0.23 nm corresponds to the (120) and (031) plane, which were present in orthorhombic AlOOH.

**Figure 2:**
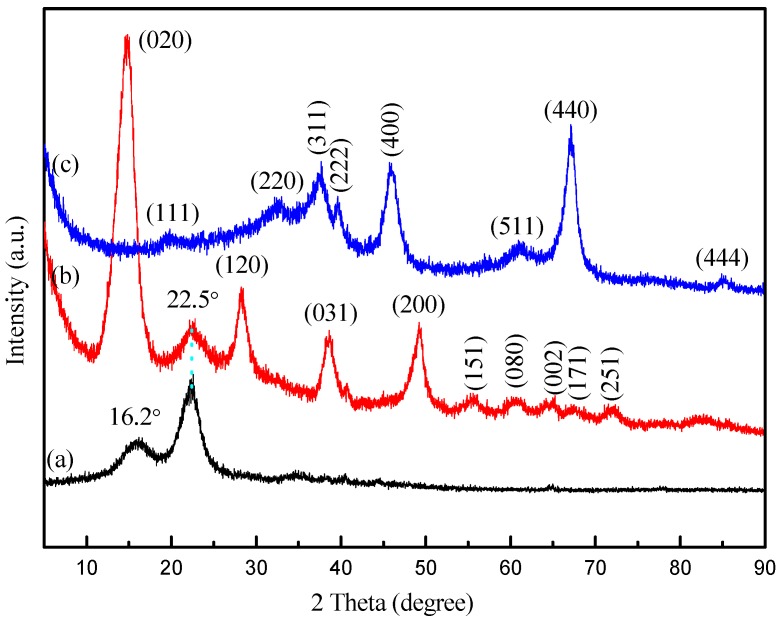
X-ray diffraction patterns of (**a**) CNF; (**b**) CNFA composite aerogel; and (**c**) CNFA heated at 900 °C under air atmosphere for 2 h in muffle.

Figure 2 shows the XRD diffractogram of (a) CNF; (b) CNFA composite aerogel. As shown in Figure 2a, there were two major diffraction peaks at 16.2° and 22.5° observed for the CNF, which could be indexed to (110) and (200) planes characteristic of native cellulose (JCPDS NO. 50-2241) [24]. In the case of CNFA composites in Figure 2b, the peaks of CNF still exist, revealing that the CNF didn’t change their structure after the hydrothermal treatment. Furthermore, nine extra diffraction peaks of CNFA were observed at 14.3°, 28.2°, 38.5°, 49.1°, 55.7°, 60.6°, 64.6°, 67.6°, and 72.1° correspond to the planes of (020), (120), (031), (200), (151), (080), (002), (171), and (251), which could be indexed to the orthorhombic AlOOH (JCPDS No. 21-1307) [25]. The results indicating that the AlOOH generated. Figure 2c showed 1 g CNFA composite aerogel heated at 900 °C under air atmosphere for 2 h in muffle. It could be observed that the peaks of cellulose were disappeared, indicating that CNF decomposed completely. Eight diffraction peaks was showed at 19.45°, 31.94°, 37.60°, 39.49°, 45.86°, 60.90°, 67.03°, and 85.02° correspond to the planes of (111), (220), (311), (222), (400), (511), (440), and (444), which could be indexed to the cubic Al_2_O_3_ (JCPDS No. 10-0425). The results were consistent with the reaction (1). The residuum was weighted as 0.2463 g which could conclude that the AlOOH was 28.98% concentrated in the sample.

**Figure 3:**
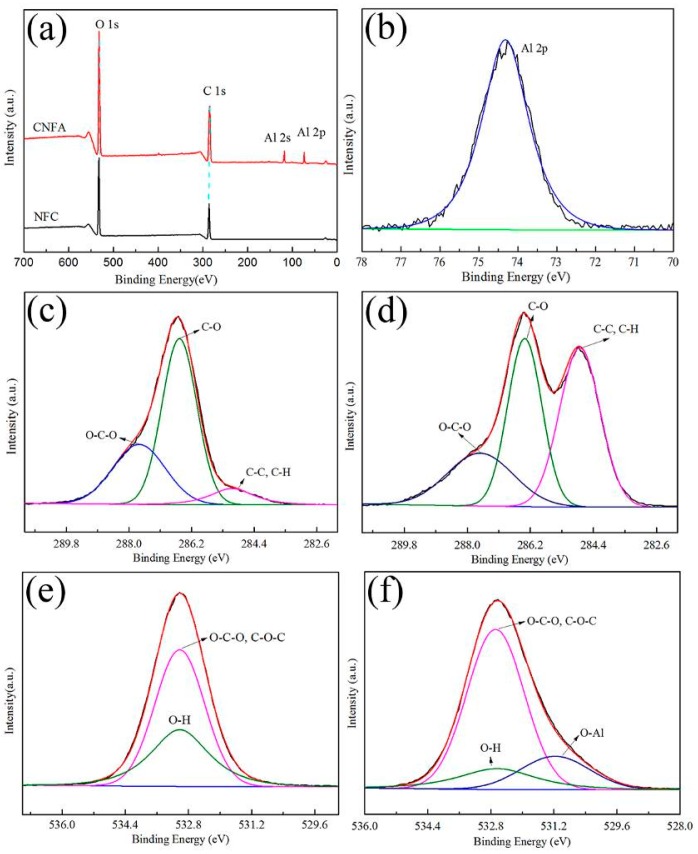
(**a**) The full scan X-ray photoelectron spectroscopy (XPS) spectra of CNF and CNFA; (**b**) high-resolution XPS spectra of Al 2p of CNFA composite aerogel; (**c**,**d**) high-resolution XPS spectra of C 1s element of CNF and CNFA; (**e**,**f**) high-resolution XPS spectra of O 1s element of CNF and CNFA.

Figure 3 showed the XPS spectra of the CNF and CNFA. Figure 3a showed the full scan XPS spectra of CNF and CNFA. The wide scan spectra of the CNF exhibited two major peaks with binding energy at 286.1 eV and 532.1 eV corresponding to the C 1s and O 1s of CNF, respectively. However, in the CNFA, an additional peak was observed at a binding energy of 75 eV and 120 eV corresponding to the Al 2p and Al 2s, which confirmed the presence of AlOOH in the CNFA composite. For understanding the chemical form of the CNF and CNFA, detailed scans of specific regions of key elements (C 1s and O 1s) and extra element (Al 2s and Al 2p) were carried out and were shown in Figure 3b–f. The binding energies of Al 2p appeared at 74.3 eV (Figure 3b), which could be well attributed to the Al element. According to the above results, it can be deduced that AlOOH particles have been successfully produced in CNFA. Figure 3c showed three peaks that were observed at 284.9 eV, 286.3 eV, and 287.9 eV in the high-resolution spectra of C 1s in CNF. It indicates the presence of C–C, C–O, and O–C–O bounds. Upon hydrothermal reaction, the high-resolution C 1s spectrum of CNFA (Figure 3d) showed the similar results to that of CNF. The same peaks located in the same places. The intensity differences observed in the peak at 284.9 eV was attribute to C–C bound. The results may be due to some degradation during hydrothermal treatment. Two peaks, which were attributed to O–H and O–C–O & C–O–C, were observed at 532.8 eV in the high-resolution spectra of O 1s in CNF (Figure 3e). Another peak was observed at 531.1 eV in the high-resolution spectra of O 1s in CNFA except the two peaks in the high-resolution spectra of O 1s in CNF at the same location (Figure 3f). The additional peak could be attributed to the O–Al. This further confirms the presence of AlOOH in the CNFA.

**Figure 4:**
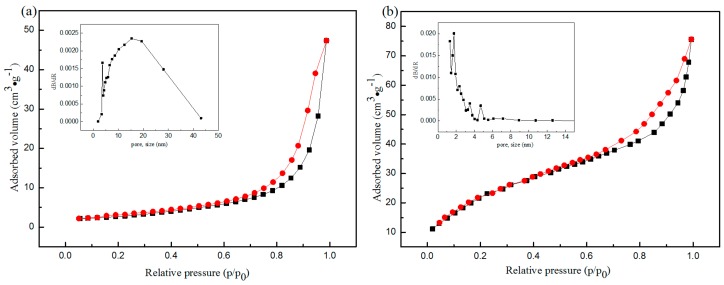
The N_2_ adsorption/desorption isotherms of (**a**) CNF and (**b**) CNFA aerogels and corresponding pore size distribution derived from the adsorption branch.

The N_2_ adsorption/desorption isotherms of CNF and CNFA aerogels displayed a direct hysteresis loop, shown in Figure 4, belonging to type IV (according to IUPAC classification). The results reflected the presence of mesoporous structure in the samples. The corresponding pore size distribution of the samples was calculated by the BJH method and was illustrated in the insert of Figure 4. The CNFA showed smaller pore diameter (dominated in about 2 nm) than CNF (dominated about 15 nm), but it also exhibited a sharper and more concentrated peak. The porosity detailed data of the CNF and CNFA was summarized in Table 1. It could be seen that the specific surface of CNFA is 6 times higher than the CNF, and the average pore diameter of CNFA was five times higher than the CNF.

**Figure 5:**
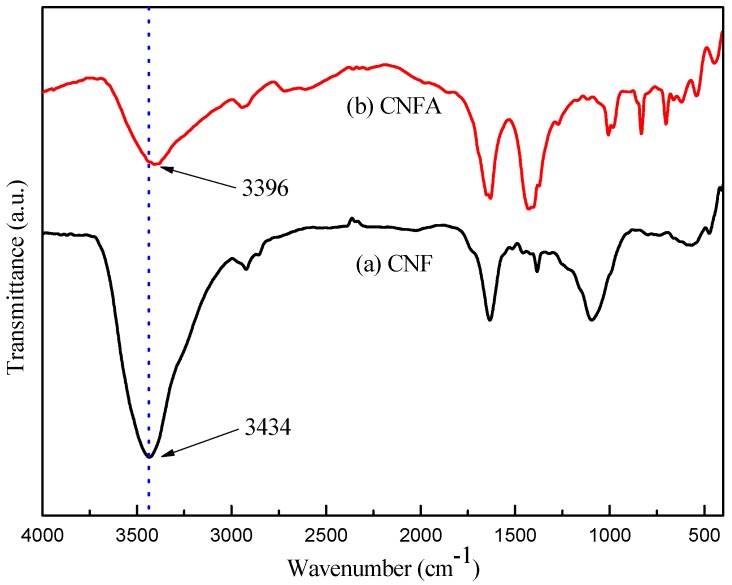
FT-IR spectra of (**a**) CNF and (**b**) CNFA.

Figure 5 showed the FT-IR spectra of the CNF and CNFA. As showed in Figure 5a, a broad and strong absorption band in the region at 3434 cm^−1^ was attributed to stretching vibrations of O-H band in cellulose. Comparing with CNFA, the peak was weak at 3396 cm^−1^ was shifted to lower wavenumbers, indicating a strong interaction between the hydroxyl groups of CNF and AlOOH through bond. This strong interaction led to AlOOH was wrapped in CNF or adhered to the surface of them.

**Figure 6:**
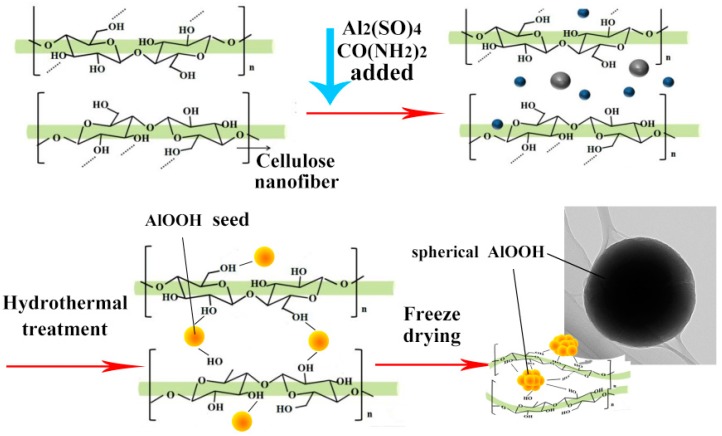
Schematic illustration of the formation process of spherical AlOOH wrapped in CNF or adhered to CNF.

Figure 6 showed the formation mechanism of the spherical AlOOH wrapped in CNF or adhered to the surface of CNF. A possible mechanism could be inferred from the interaction of hydrogen bond between AlOOH and the surface of CNF. As we all know that there are plenty of hydroxyl groups on the surface of cellulose, during the hydrothermal processing, AlOOH was generated and attracted by the interaction of the hydrogen bond between the hydroxyl groups on CNF and the hydroxyl groups on the surface of AlOOH. This further helped in the generation of the AlOOH nuclei on the surface of the CNF. As the reaction proceeded, the nuclei would grow and form spherical AlOOH on the surface of the CNF or adhered to CNF. Therefore, CNF could be close to spherical AlOOH particles and wrap it like a net act as a shell or adhered to CNF. Subsequently, freeze-drying occurred to sublime the liquid into gas phase. The lightweight CNFA aerogel was fabricated.

**Figure 7:**
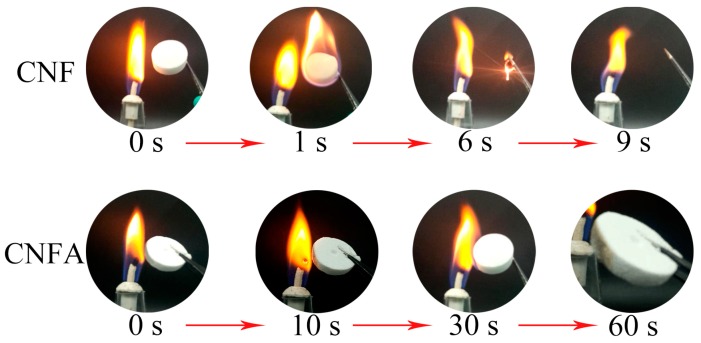
The combustion test of CNF and CNFA aerogel.

The combustion tests of CNF and CNFA aerogel by an alcohol burner were studied. Figure 7 presented the details. CNF aerogel was easy to ignite and burned up quickly, indicating that the CNF was poor in flame resistance. It is a typical combustion behavior for normal cellulose. On the other hand, it can be observed that the as-prepared CNFA composite aerogels exhibited excellent fire resistance when exposed to the flame. CNFA composite aerogels were not burnt immediately. The sample was still preserved well in 60 s and kept an integrated shape. There was only a yellow color appearing at the edge. It was undoubtedly demonstrated that the existence of AlOOH particles could improve fire resistant properties of CNF aerogel. The flame retardant properites of CNFA was much better than the reports of Yuan et al. (self-extinguishing once the flame was removed after cellulose/aluminum hydroxide nanocomposite aerogels had been burning for 3 s) [26] and Han et al. (the magnesium hydroxide nanoparticles in cellulose gel composite aerogels still remained to some extent after 10 s of burning, and the residual length did not change notably among) [23].

**Figure 8:**
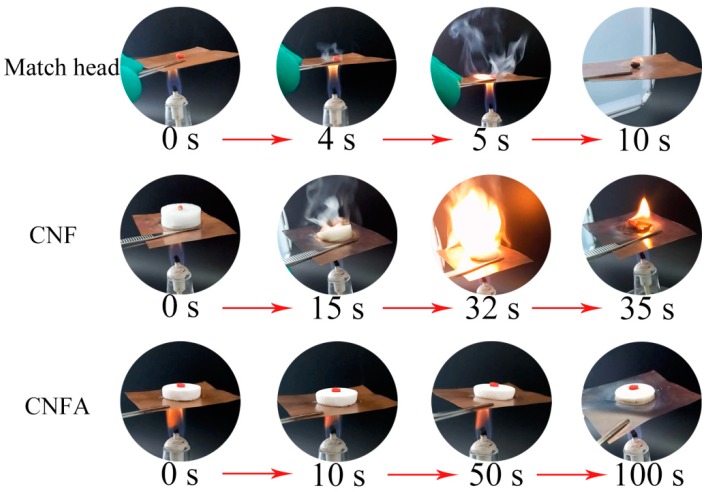
The thermal insulation test of CNF and CNFA aerogel.

In order to investigate the thermal insulation properties of CNF and CNFA, a facile simulation experiment was studied in Figure 8. In this case, the match head was selected as an inflammable material. The match head was put on a metal sheet (copper as the mound) in order to simulate the scene of fire, then an alcohol lamp was heated. It could be observed that the match head started to smoke after 4 s and burnt in 5 s. When a CNF aerogel was between the copper sheet and match head, the aerogel acted as a thermal insulating layer. It was seen that the CNF aerogel started to smoke after 15 s, burn at 32 s, turned to ashes in 35 s, the match head was not ignited until the burning of the CNF aerogel. The thermal conductivity and diffusivity of the CNF aerogel were measured and the results were 0.0477 W·m^−1^·k^−1^ and 0.621 mm^2^·s^−1^, respectively. When CNFA aerogel was between the copper and match head, the match head did not burn before 100 s, the shape of aerogel kept its complete integrity. The thermal conductivity and diffusivity of the CNFA aerogel were 0.0385 W·m^−1^·k^−1^ and 0.341 mm^2^·s^−1^, respectively. The results suggested that the CNFA was an excellent material for thermal insulation. The thermal conductivity was similar to the nano-fibrillated cellulose-zeolites aerogel reported by Bendahou et al. (0.033 W·m^−1^·k^−1^) [27].

## 4. Conclusions

Cellulose nanofiber/AlOOH aerogel was successfully prepared through a hydrothermal method. The average diameter of CNF was 56.3 nm before the reaction. With the hydrothermal method proceeding, a spherical AlOOH particle with average diameter of 0.5 μm was generated and was either wrapped by CNF or adhered to them. Their flame retardant and thermal insulation properties were investigated. CNFA composite aerogel exhibited superior flame retardant and thermal insulation properties through the flammability test, indicating that the as-prepared composite aerogel would have a promising future in the application of some important areas such as protection of lightweight construction materials.

## Figures and Tables

**Table 1:** Texture properties of CNF and CNFA.

Sample	SBET (m^2^·g^−1^)	Average Pore Diameter (nm)	Vtotal (cm^3^·g^−1^)
CNF	10.1	29.3	0.073
CNFA	66.9	5.57	0.093
